# Supplementary material for: Cost-effectiveness and budget impact analysis of PPV23 vaccination for the Malaysian Hajj pilgrims
Source: PLoS One. 2022 Jan 24;17(1):e0262949. doi: 10.1371/journal.pone.0262949 (PMC8786116; doi:10.1371/journal.pone.0262949)

PLOS ONE

Cost-effectiveness and budget impact analysis of PPV23 vaccination for the Malaysian Hajj pilgrims

--Manuscript Draft--

**Manuscript Number:**	PONE-D-21-27712
**Article Type:**	Research Article
**Full Title:**	Cost-effectiveness and budget impact analysis of PPV23 vaccination for the Malaysian Hajj pilgrims
**Short Title:**	Cost-effectiveness and budget impact analysis of PPV23 vaccination
**Corresponding Author:**	Farhana Aminuddin, Ph.D.<br>Ministry of Health Malaysia: Kementerian Kesihatan Malaysia<br>Shah Alam, Selangor Darul Ehsan MALAYSIA
**Keywords:**	Cost-effectiveness analysis; budget impact; PPV23; pneumococcal disease; Hajj pilgrims
**Abstract:**	The potential occurrence of disease outbreaks during the hajj season is of great concern due to extreme congestion in a confined space. This promotes the acquisition, spread and transmission of pathogenic microorganisms and pneumococcal disease are one of the most frequent infections among Hajj pilgrims. This study aimed to assess the cost-effectiveness and budget impact of introducing the PPV23 to Malaysian Hajj pilgrims. A decision tree framework with a 1-year cycle length was adapted to evaluate the cost-effectiveness of a PPV23 vaccination program with no vaccination. The cost information was retrieved from the Lembaga Tabung Haji Malaysia (LTH) database. Vaccine effectiveness was based on the locally published data and the disease incidence specifically related to Streptococcus pneumoniae was based on a literature search. Analyses were conducted from the perspective of the provider: Ministry of Health and LTH Malaysia. The incremental cost-effectiveness ratios (ICER), cases averted, and net cost savings were estimated. Findings from this study showed that PPV23 vaccination for Malaysian hajj pilgrims was cost-effective. The PPV23 vaccination programme has an ICER of MYR -449.3 (US$-110.95) per case averted. Based on the national threshold value of US$6,200-US$8,900 per capita, the base-case result shows that introduction of the PPV23 vaccine for Malaysian Hajj pilgrims is very cost-effective. Sensitivity analysis revealed parameters related to annual incidence and hospitalised cost of septicemia and disease without vaccination as the key drivers of the model outputs. Compared with no vaccination, the inclusion of PPV23 vaccination for Malaysian Hajj pilgrims was projected to result in a net cost saving of MYR59.6 million and 109,996 cases averted over 5 years period. The PPV23 vaccination program could substantially offer additional benefits in reducing the pneumococcal disease burden and healthcare cost. This could be of help for policymakers to consider the implementation of PPV23 vaccination for Malaysian performing hajj.
**Order of Authors:**	Farhana Aminuddin, Ph.D.
	Nor Zam Azihan Mohd Hassan
	Nur Amalina Zaimi
	Mohd Shaiful Jefri Mohd Nor Sham Kunusagaran
	Mohd Shahri Bahari
	Mohd Ridzwan Shahari
**Additional Information:**	
**Question**	**Response**
**Financial Disclosure**<br><br>Enter a financial disclosure statement that	This study was supported/ funded by the Ministry of Health Malaysia (NMRR-17-471-34944). The funder had no role in study design, data collection and analysis, or preparation of the manuscript.

Powered by Editorial Manager® and ProduXion Manager® from Aries Systems Corporation

describes the sources of funding for the work included in this submission. Review the submission guidelines for detailed requirements. View published research articles from *PLOS ONE* for specific examples.<br><br>This statement is required for submission and **will appear in the published article** if the submission is accepted. Please make sure it is accurate.<br><br>**Unfunded studies**<br>Enter: *The author(s) received no specific funding for this work.*<br><br>**Funded studies**<br>Enter a statement with the following details:<br>• Initials of the authors who received each award<br>• Grant numbers awarded to each author<br>• The full name of each funder<br>• URL of each funder website<br>• Did the sponsors or funders play any role in the study design, data collection and analysis, decision to publish, or preparation of the manuscript?<br>• **NO** - Include this sentence at the end of your statement: *The funders had no role in study design, data collection and analysis, decision to publish, or preparation of the manuscript.*<br>• **YES** - Specify the role(s) played.<br><br>* typeset	
**Competing Interests**<br><br>Use the instructions below to enter a competing interest statement for this submission. On behalf of all authors, disclose any competing interests that could be perceived to bias this work—acknowledging all financial support and any other relevant financial or non-financial competing interests.<br><br>This statement is required for submission and **will appear in the published article** if the submission is accepted. Please make sure it is accurate and that any funding	The authors have declared that no competing interests exist.

sources listed in your Funding Information later in the submission form are also declared in your Financial Disclosure statement.<br><br>View published research articles from *PLOS ONE* for specific examples.<br><br>**NO authors have competing interests**<br><br>Enter: *The authors have declared that no competing interests exist.*<br><br>**Authors with competing interests**<br><br>Enter competing interest details beginning with this statement:<br><br>*I have read the journal's policy and the authors of this manuscript have the following competing interests: [insert competing interests here]*<br><br>* typeset	
**Ethics Statement**<br><br>Enter an ethics statement for this submission. This statement is required if the study involved:<br><br>• Human participants<br>• Human specimens or tissue<br>• Vertebrate animals or cephalopods<br>• Vertebrate embryos or tissues<br>• Field research<br><br>Write "N/A" if the submission does not require an ethics statement.<br><br>General guidance is provided below. Consult the submission guidelines for detailed instructions. **Make sure that all information entered here is included in the Methods section of the manuscript.**	Ethical approval for this study was obtained from the Medical Research & Ethics Committee (MREC), Ministry of Health Malaysia: KKM/NIHSEC/800-6/2/5 JLD.2(17).

Powered by Editorial Manager® and ProduXion Manager® from Aries Systems Corporation

**Format for specific study types**<br><br>**Human Subject Research (involving human participants and/or tissue)**<br>• Give the name of the institutional review board or ethics committee that approved the study<br>• Include the approval number and/or a statement indicating approval of this research<br>• Indicate the form of consent obtained (written/oral) or the reason that consent was not obtained (e.g. the data were analyzed anonymously)<br><br>**Animal Research (involving vertebrate animals, embryos or tissues)**<br>• Provide the name of the Institutional Animal Care and Use Committee (IACUC) or other relevant ethics board that reviewed the study protocol, and indicate whether they approved this research or granted a formal waiver of ethical approval<br>• Include an approval number if one was obtained<br>• If the study involved *non-human primates*, add *additional details* about animal welfare and steps taken to ameliorate suffering<br>• If anesthesia, euthanasia, or any kind of animal sacrifice is part of the study, include briefly which substances and/or methods were applied<br><br>**Field Research**<br><br>Include the following details if this study involves the collection of plant, animal, or other materials from a natural setting:<br>• Field permit number<br>• Name of the institution or relevant body that granted permission	
**Data Availability**<br><br>Authors are required to make all data underlying the findings described fully available, without restriction, and from the time of publication. PLOS allows rare exceptions to address legal and ethical concerns. See the PLOS Data Policy and FAQ for detailed information.	Yes - all data are fully available without restriction

A Data Availability Statement describing where the data can be found is required at submission. Your answers to this question constitute the Data Availability Statement and **will be published in the article**, if accepted.<br><br>**Important:** Stating 'data available on request from the author' is not sufficient. If your data are only available upon request, select 'No' for the first question and explain your exceptional situation in the text box.<br><br>Do the authors confirm that all data underlying the findings described in their manuscript are fully available without restriction?	
**Describe where the data may be found in full sentences. If you are copying our sample text, replace any instances of XXX with the appropriate details.**<br><br>• If the data are **held or will be held in a public repository**, include URLs, accession numbers or DOIs. If this information will only be available after acceptance, indicate this by ticking the box below. For example: *All XXX files are available from the XXX database (accession number(s) XXX, XXX.*).<br>• If the data are all contained **within the manuscript and/or Supporting Information files**, enter the following: *All relevant data are within the manuscript and its Supporting Information files.*<br>• If neither of these applies but you are able to provide **details of access elsewhere**, with or without limitations, please do so. For example:<br><br>*Data cannot be shared publicly because of [XXX]. Data are available from the XXX Institutional Data Access / Ethics Committee (contact via XXX) for researchers who meet the criteria for access to confidential data.*<br><br>*The data underlying the results presented in the study are available from (include the name of the third party*	All data generated and/ or analysed during the current study are either included in this published article or can be found in the referenced literature.

and contact information or URL).<br>• This text is appropriate if the data are owned by a third party and authors do not have permission to share the data.<br><br>* typeset	
Additional data availability information:	

Manuscript

Click here to access/download;Manuscript;CEA_PPV23_PLOS.docx

Cost-effectiveness and budget impact analysis of PPV23 vaccination for the Malaysian Hajj pilgrims

Farhana Aminuddin[1*], Nor Zam Azihan Mohd Hassan[1], Nur Amalina Zaimi[1], Mohd Shaiful Jefri Mohd Nor Sham Kunusagaran[1], Mohd Shahri Bahari[1], Mohd Ridzwan Shahari[2]

[1]Institute for Health Systems Research, National Institute of Health (NIH), Ministry of Health Malaysia, Setia Alam, Shah Alam, Selangor, Malaysia.

[2]Medical Development Division, Ministry of Health Malaysia, Complex E, Federal Government Administrative Centre, Putrajaya, Malaysia.

* Corresponding author

farhana.a@moh.gov.my

Abstract

The potential occurrence of disease outbreaks during the hajj season is of great concern due to extreme congestion in a confined space. This promotes the acquisition, spread and transmission of pathogenic microorganisms and pneumococcal disease are one of the most frequent infections among Hajj pilgrims. This study aimed to assess the cost-effectiveness and budget impact of introducing the PPV23 to Malaysian Hajj pilgrims. A decision tree framework with a 1-year cycle length was adapted to evaluate the cost-effectiveness of a PPV23 vaccination program with no vaccination. The cost information was retrieved from the Lembaga Tabung Haji Malaysia (LTH) database. Vaccine effectiveness was based on the locally published data and the disease incidence specifically related to *Streptococcus pneumoniae* was based on a literature search. Analyses were conducted from the perspective of the provider: Ministry of Health and LTH Malaysia. The incremental cost-effectiveness ratios (ICER), cases averted, and net cost savings were estimated. Findings from this study showed that PPV23 vaccination for Malaysian hajj pilgrims was cost-effective. The PPV23 vaccination programme has an ICER of MYR -449.3 (US$-110.95) per case averted. Based on the national threshold value of US$6,200-US$8,900 per capita, the base-case result shows that introduction of the PPV23 vaccine for Malaysian Hajj pilgrims is very cost-effective. Sensitivity analysis revealed parameters related to annual incidence and hospitalised cost of septicemia and disease without vaccination as the key drivers of the model outputs. Compared with no vaccination, the inclusion of PPV23 vaccination for Malaysian Hajj pilgrims was projected to result in a net cost saving of MYR59.6 million and 109,996 cases averted over 5 years period. The PPV23 vaccination program could substantially offer additional benefits in reducing the pneumococcal disease burden and healthcare cost. This could be of help for policymakers to consider the implementation of PPV23 vaccination for Malaysian performing hajj.

Keywords: Cost-effectiveness analysis, budget impact, PPV23, pneumococcal disease, Hajj pilgrims

Introduction

Millions of Muslims from more than 180 countries converge in Saudi Arabia to perform Hajj each year [1]. The overcrowding and extreme congestion due to confined spaces present a significant challenge to the health of pilgrims as they are more susceptible to acquiring and spreading infectious diseases, likes respiratory tract infections. Among the respiratory illnesses, Community-acquired pneumonia (CAP) being the primary cause of critical illness and the leading cause of hospital admission among pilgrims [2-4]. A broad array of pathogens are corresponding to respiratory disorders, with *Streptococcus pneumoniae* being the most common isolated from patients [5]. This pathogen could also cause diseases with varied in severity from otitis media to CAP, through invasive pneumococcal diseases (IPD) such as meningitis and septicemia. Individuals with chronic diseases, the elderly and infants are at risk of severe infection [6-7].

A substantial proportion of Hajj pilgrims who arrived in Saudi are older adults aged >50 and >65 years with more than 50% and 25%, respectively [8]. Many Hajj pilgrims also have underlying medical conditions such as chronic lung disease, chronic heart disease, diabetes or other chronic conditions [4, 9-10]. Hajj pilgrims, especially those of extreme age or with pre-existing medical conditions are at risk of pneumococcal disease, thus, consideration should be given to vaccinating high-risk pilgrims against pneumococcal disease. The ideal vaccine profile would be one that has an impact on pathogen transmission through a reduction in the amount of bacterial acquisition. The likely candidate, 23-valent polysaccharides pneumococcal vaccine (PPV23) was found to be effective and reported to reduce the risk of CAP and IPD in older adults ≥65 years [11-12]. Yet, the cost-effectiveness of PPV23 among hajj pilgrims is unknown.

Studies on economic evaluations such as the cost-effectiveness of pneumococcal vaccines among adults have been conducted in many countries with diverse outcomes [13-14]. The PPV23 was found to be cost-effective among the elderly aged ≥60 years in several studies. According to Wolff et al [15], the PPV23 was reported to significantly reduce the burden of pneumococcal disease and is likely to be cost-effective for 75 year-olds in a Swedish setting. Besides, PPV23 was reported as cost-effective over the PCV13 among the elderly (≥65) in the Netherlands [16]. A previous study has also analysed the cost-effectiveness of PPV23

vaccination in different age groups, which PPV23 has been found most cost-effective in older adults aged ≥60 years [17]. The results, however, are difficult to translate to the Hajj pilgrims given the diverse population and unique event.

Respiratory tract infection was reported during the hajj season, which accounted for 74% of all medical illnesses [18] and 39% of all patients being reported hospitalised due to pneumonia [19]. For this, respiratory tract infection continues to exert a health and economic burden on a health system of a country. As a country with a majority of Muslim population, thousands of Malaysian Muslims attended hajj each year. A study conducted by Deris et al. [20] reported that 90% of Malaysian Hajj pilgrims had at least one respiratory symptom. Another study conducted by Balkhy et al. [21] suggested that one in three pilgrims was estimated to experience respiratory symptoms. While healthcare-related costs of hospitalisation, medication and emergencies in Saudi are all covered by the Malaysian government, the most cost-effective and efficacious vaccine to prevent pneumococcal disease should be recommended and implemented.

Many countries are evolved in developing or have developed recommendations for pneumococcal vaccination for Hajj pilgrims including the Gulf countries (Qatar, Kuwait, United Arab Emirates, Bahrain and Oman), the USA, certain European countries (the UK, France and Germany) and Malaysia [22]. To date, pneumococcal vaccination is not officially recommended for the Hajj pilgrims by the Saudi Arabia authorities; the government of Saudi Arabia has only mandated meningococcal meningitis vaccination for all Hajj pilgrims [23]. The policy on pneumococcal vaccination remains optional in many countries including Malaysia. Decisions about implementation of pneumococcal vaccine intervention for hajj pilgrims requires a careful assessment at the country level, by considering the pneumococcal disease incidence, hospitalisation rates due to pneumococcal disease in Saudi, vaccine effectiveness and vaccination cost. Hence, this study was carried out to assess the cost-effectiveness and budget impact of introducing the PPV23 vaccination strategy versus no vaccination from the perspective of the Malaysian government. The findings from this study will provide evidence for policymakers if PPV23 is to be considered as an additional immunization programme for Malaysian hajj pilgrims.

Materials and Methods

Model structure

A decision tree model was adapted from Aljunid et al. [24] and developed with modification to determine the cost-effectiveness of introducing a PPV23 vaccination strategy for Malaysian performing hajj. Costs, measured in MYR, and health effects, refer to cases averted were assigned to each health state in the model. The base case model was applied to the 2017 Hajj pilgrims cohort (N = 40,837) and analysis was based on a one-year time horizon. The health states built into the model are shown in Fig 1 and the model considered the following disease categories; meningitis, pneumonia, otitis media, septicemia, sinusitis and death. All causes of pneumococcal disease were at risk of death. The impact of vaccination on costs was broken down into costs related to vaccination implementation and costs averted due to reduced healthcare burden. All costs were valued in Malaysian Ringgit in 2017 with a 3% discounted rate was used, where applicable. The health effects were quantified as cases averted due to vaccination. The analysis was performed from the provider's perspective.

Fig 1. A decision tree model, illustrating the two alternatives for PPV23 vaccination with the status quo of no vaccination. The spectrum of pneumococcal infections included: meningitis, pneumonia, otitis media, septicemia, sinusitis and death.

Epidemiological and disease data

Model inputs for epidemiological data including the health status background and disease burden of 2017 Malaysian Hajj pilgrims were obtained from the Lembaga Tabung Haji (LTH) Malaysia database, LTH-THIS. However, unavailable epidemiological and incidence of pneumococcal disease studies in Malaysia necessitated the use of data from other countries. Therefore, the probability of each pneumococcal disease incidence was obtained from the published literature, which was confined to a population group of hajj pilgrims or the elderly (Table 1).

Table 1. Probabilities and estimates associated with pneumococcal disease.

Variables	Base-case value	Range for sensitivity analysis$^{\Psi}$	Distribution	Source
Incidence rates of pneumococcal disease:				
With vaccination	0.120	0.090-0.150	Beta	Calculated from [25]
Without vaccination	0.620	0.465-0.775	Beta	Calculated from [25]
Incidence rates of pneumococcal outcome:				
Meningitis	0.007	0.005-0.009	Beta	[26]
Pneumonia	0.230	0.173-0.288	Beta	[2]
Hospitalised	0.017	0.013-0.021	Beta	Calculated from [24]
Otitis media	0.200	0.150-0.250	Beta	[27]
Septicemia	0.250	0.188-0.313	Beta	[28]
Hospitalised	0.059	0.044-0.074	Beta	Calculated from [24]
Sinusitis	0.100	0.075-0.125	Beta	[29]
Number of Malaysian Hajj pilgrims$^{\Psi\Psi}$	40,837	30,628- 51,046	Normal	LTH-THIS

$^{\Psi}$A range of ±25% of the base-case value was used when reported data were not available.
$^{\Psi\Psi}$Number of Malaysian hajj pilgrims visited Saudi Arabia in 2017.

Vaccine effectiveness

The PPV23 vaccine effectiveness has been derived based on available local data that could be applied in our model. Vaccine effectiveness against pneumococcal disease was taken from a prospective cohort study conducted in 2015 by the Institute for Medical Research (IMR) Malaysia of 1,000 individuals aged 50 years and older performing hajj [25]. The study has reported that PPV23 vaccine effectiveness was 87.5%. For this, we used the vaccine effectiveness of ~88% and applied it in the base-case analysis.

Costs estimation

This study considered different methods to estimate the outpatient and inpatient costs given the lack of data availability such as individual patient's medical visits and admission records. Outpatient and inpatient care costs included the cost of facility rentals, vehicle rentals, utilities, drugs, and staff allowances used during pilgrim's visits to the clinic and hospital admission. A top-down costing approach for outpatient was applied to calculate the operational costs incurred by the Ministry of Health (MOH) and LTH Malaysia for the management and treatment of pneumococcal disease based on actual expenditures in 2017 (Table 2). The costs for both clinics and hospitals were estimated using different apportion techniques; the total costs of the vehicles, electricity, water, salary, and petrol were equally distributed (50%:50%) between clinics and hospitals. While 60%:40% were allocated for drugs and equipment between clinics and hospitals, respectively. The apportionment of resources was by the experts' opinion based on the pattern of health care utilisation in clinics and hospitals. The cost of outpatient care was calculated based on the average cost of treatment per pilgrims per visit.

The cost of hospitalisation for Malaysian Hajj Pilgrims in Saudi Arabia was adapted from the Malaysian Diagnosis-related Group Case-mix Systems (My-DRG) costing data under a category of Severity 1 Level as a baseline to estimate the minimum cost of hospitalisation in Saudi Arabia (Table 3). This is due to limitations of data such as facilities floor space and the admission ratio of pneumococcal-related disease to other diseases. The costing data is then adjusted to the Purchasing Power Parity (PPP) per capita ratio between Saudi Arabia (US$47,309.132) and Malaysia (US$26,661.188) in 2017 [30].

Because there is no national vaccine cost data available, the PPV23 vaccine's unit price was adopted and derived from Mo et al [31]. All costs of vaccination were calculated in Malaysian Ringgit (MYR) for the year 2017 (USD 1 = RM4.30) after being adjusted to an average annual inflation rate of 3%. For this, the cost of vaccination used for the analysis was estimated at RM130.80 per dose. The costs of pneumococcal disease treatment are tabulated in Table 4.

Table 2. Costs estimate per year for outpatient care to treat pneumococcal disease based on actual expenditures in 2017.

	Outpatient care (MYR)
Rentals	
Clinics	3,074,400
Vehicles	216,978
Utility bills	
Electricity	54,000
Water	39,600
Drugs & consumables	
Purchasing cost in Malaysia	1,632,000
Delivery cost to Saudi Arabia	138,000
Purchasing cost in Saudi Arabia	90,000
Salary & allowances	1,128,731.50
Others	
Petrol for vehicles	5,000
Patient's foods	0
**Total costs**	**6,378,709.50**
Total no. of visits	54,495
Total cost per visit	117.05
Frequency of visits per person$^{\Psi}$	3
**Outpatient**	
Cost per person	**351.15**

$^{\Psi}$An average of 3 visits per one treatment per person was assumed, which was based on LTH expert opinion.

Table 3. Cost estimations for inpatient care to treat pneumococcal disease in 2017 based on My-DRG.

Inpatient care (per admission)	Malaysia (DRG-Severity 1) (MYR)	Saudi (MYR)$^{\$}$
Pneumonia	5,435.88	9,944.92
Septicemia	9,338.95	17,085.57
Meningitis	11,920.75	21,808.96

$^{\$}$Estimated based on PPP per capita ratio between Malaysia and Saudi in 2017 [30] and a 3% annual inflation rate was applied.

Table 4. The costs of vaccine per dose and pneumococcal disease.

Variables	Base-case estimate (MYR)	Range for sensitivity analysis$^{\Psi}$	Distribution	Source
Cost of vaccination	130.80	98.10-163.50	Gamma	[**Error! Reference source not found.**]
Cost of treatment				
Meningitis	21,808.96	16,356.72- 27,261.20	Gamma	My-DRG
Pneumonia	9,944.92	7,458.69- 12,431.15	Gamma	My-DRG
Otitis media	351.15	263.36- 438.94	Gamma	Calculated$
Septicemia	17,085.57	12,814.18- 21,356.96	Gamma	My-DRG
Sinusitis	351.15	263.36- 438.94	Gamma	Calculated$

$Using the cost data obtained from the LTH-THIS database.

Cost-effectiveness analysis

Incremental cost-effectiveness ratios (ICERs), which are a measure of cost-effectiveness, was reported as cost per case averted. The ICERs were determined by dividing the treatment costs of pneumococcal disease (with or without PPV23 vaccination) by the incremental effectiveness (measured as cases averted) between the two interventions. ICER was reported as incremental cost per case averted. The cost savings due to vaccine prevented cases were also reported. A national cost-effectiveness threshold was used, whereby an ICER between 0.90 and 1.28 (MYR19,929 and MYR28,470 or US$6,200-US$8,900 per capita) is considered cost-effective and very cost-effective when the ICER value is below the threshold range [32].

Uncertainty analysis

One-way sensitivity analysis

Sensitivity analyses were performed to assess the influence of input parameters on the cost-effectiveness results. A one-way sensitivity analysis (vaccination vs no vaccination) was

conducted to test the robustness of the model and to find the most significant variables influencing the cost-effectiveness of the PPV23 vaccination programme. The result of the one-way sensitivity analysis is presented in a Tornado diagram. The values for each base-case model parameter were obtained from the relevant literature and a range of ±25% of the base-case values was used when reported data were not available (see Tables 1 and 4).

Probabilistic sensitivity analysis

The probabilistic sensitivity analysis was performed to evaluate the impact of the uncertainty in all of the variables simultaneously; model inputs were sampled using the Monte Carlo method with 1,000 simulations. The costs value for each fit from a gamma distribution and probabilities fit beta distribution generated from the 95% uncertainty range. While under the normal distribution, a sample pool of the hajj population was generated. The cost-effectiveness acceptability curve (CEAC) for vaccination vs no vaccination was also developed to present the probability of cost-effectiveness iterations at different WTP values.

Budget impact analysis

BIA was calculated to project a financial burden for future health expenditures if the PPV23 vaccination programme is introduced to Malaysian Hajj pilgrims compared to no vaccination. The total budget of the vaccination programme was calculated by multiplying the number of Hajj pilgrims per year by the cost of the vaccine per dose. We assumed 100% of Hajj pilgrims will be vaccinated each year, and the cost of vaccination is adjusted to the annual inflation rate of 3%. The treatment costs averted due to the PPV23 vaccination programme was presented to obtain the net incremental cost of the programme.

Results

Base case analysis

The results from the cost-effectiveness analyses of PPV23 vaccination are presented in Table 5. It was estimated that vaccination with a PPV23 would prevent 35,937 pneumococcal-related cases compared with no vaccination strategy for the Malaysian Hajj cohort of 40,837 in 2017. The PPV23 vaccination would incur a cost of MYR8.8 million while the Malaysian health system would incur a total cost of MYR18 million for the study cohort due to pneumococcal disease without vaccination in place. In terms of cost-effectiveness, the PPV23 vaccination programme has an ICER of MYR -449.3 per case averted compared with a status quo of no vaccination. Based on the national threshold value of US$6,200-US$8,900 per capita, the base-case result shows that introduction of the PPV23 vaccine for Malaysian Hajj pilgrims is very cost-effective.

Table 5. Cost-effectiveness analysis of the PPV23 vaccination programme in a cohort of Malaysian Hajj pilgrims.

Variable	No vaccination	Vaccination	Incremental events or costs (averted)	Incremental events or costs (incurred)
Cost of vaccination	-	5,341,479.60	-	5,341,479.60
Cost of treatment				
Meningitis	3,865,368.21	748,114.50	3,117,143.74	-
Pneumonia	2,994,626.57	579,605.14	46,703,880.25	-
Otitis media	1,778,359.21	344,157.90	1,433,991.25	-
Septicemia	8,472,228.71	1,639,786.20	87,215,427.75	-
Sinusitis	888,754.30	172,078.96	716,995.61	-
Total	17,999,337.00	8,825,222.30	10, 840,264.28	-
Effects (case averted)	15,518	35,937	-	20,419
ICER (per case averted)	-	-449.30	-	-

All costs were measured in Malaysian Ringgit (MYR)

Uncertainty analyses

One-way sensitivity analysis

The results of one-way sensitivity analysis are shown in the tornado diagram (Fig 2), which graphically represent how variations in each parameter affect the outcome (ICERs). The

ICERs of PPV23 vaccination versus a no vaccination strategy ranged from MYR-533.63 to -331.77. The annual incidence of getting the disease without vaccination and septicemia are the most influential parameters in the model output. Another parameter with a remarkable impact on results was the PPV23 vaccination cost. These variables were also identified as the key drivers of the model outputs. The parameters, such as the treatment cost of meningitis, otitis media and sinusitis, only have medium and small impacts. The results demonstrated that this model was also sensitive to the cost of the vaccine and the probabilities of getting the disease with vaccination, which showed a positive relationship.

Fig 2. Tornado chart presenting a one-way sensitivity analysis for PPV23 vaccination. The tails of each bar represent the differences in ICERs with low (left) and high (right) parameter values. The maximum and minimum values for each variable are presented in brackets. Prob: Probability; EV: Expected value.

Probabilistic sensitivity analysis

A Monte Carlo simulation with 1,000 iterations was done by comparing the PPV23 vaccination programme with no vaccination (Fig 3). This shows that the cost-effectiveness probabilities represented as a cloud of points lie in the South Eastern (SE) quadrant plane (97%) after considering all the uncertainties in the analysis. This corresponds that the introduction of the PPV23 vaccine provides additional health benefits (incremental effectiveness increases) at a lower cost (incremental cost decreases) despite the variation of parameters. Fig 4 illustrates the cost-effectiveness acceptability curve to assess the threshold of willingness-to-pay (WTP) for the PPV23 vaccine in Malaysia. It demonstrates that vaccination was more effective compared to no vaccination throughout various WTP threshold values up to MYR 240,000.

Fig 3. Scatter plot showing results of Monte Carlo probabilistic sensitivity analysis for PPV23 vaccination programme vs no vaccination.

Fig 4. Cost-effectiveness acceptability curve showing the probability that vaccination is cost-effective compared to no vaccination over a range of values.

Budget impact analysis

The budget impact analysis of the PPV23 vaccine was projected as tabulated in Table 6. The total expenditure on vaccinating a cohort of Hajj pilgrims for PPV23 vaccination during the year 2021-2025 was MYR34.7 million. While in the current scenario, without PPV23 vaccination, Malaysia's healthcare spending on pneumococcal disease among Hajj pilgrims for a 5-year (2021-2025) total is estimated to be MYR117.1 million. In this base case analysis, the number of cases averted grows over time due to the progressive increase in the number of Hajj pilgrims annually. Inclusion of PPV23 vaccination for a cohort of Hajj pilgrims will result in a net cost saving of MYR59.6 million and 110,996 cases averted over 5 years (2021-2025) period after discounting. From these findings, the introduction of the PPV23 vaccine for Malaysian Hajj pilgrims will affect the healthcare budget by almost 50% cost reduction yearly.

Table 6. Budget Impact Analysis of PPV23 Vaccination.

	2017	2018	2019	2020	2021	2022	2023	2024	2025	Total (5 years results)[ΨΨ]
Number of Hajj pilgrims[Ψ]	40,837	41,409	41,988	42,576	43,172	43,777	44,390	45,011	45,641	
No vaccination										
Number of cases	25,319	25,673	26,033	26,397	26,767	27,142	27,522	27,907	28,298	
Cost of treatment (MYR in million)	18.0	18.8	19.6	20.5	21.4	22.4	23.4	24.4	25.5	117.1
Vaccination										
Number of pneumococcal-related cases	4,900	4,969	5,039	5,109	5,181	5,253	5,327	5,401	5,477	
Cost of vaccination/ dose (MYR)	130.8	134.7	138.8	142.9	147.2	151.6	156.2	160.9	165.7	
Cost of vaccination (MYR in million)	5.3	5.6	5.8	6.1	6.4	6.6	6.9	7.2	7.6	34.7
Cost of treatment (MYR in million)	3.5	3.6	3.8	4.0	4.1	4.3	4.5	4.7	4.9	22.5
Total cost (vaccination + treatment) (MYR in million)	8.8	9.2	9.6	10.1	10.5	10.9	11.4	11.9	12.5	57.2
Number of cases averted	20,419	20,704	20,994	21,288	21,586	21,888	22,195	22,506	22,821	110,996
Net cost-saving (MYR in million)	9.2	9.6	10.0	10.4	10.9	11.4	11.9	12.4	13.0	59.6

The cost of vaccination was adopted from Mo et al [31] and adjusted to annual inflation rate of 3%.
[Ψ]Number of Hajj pilgrims was calculated based on Malaysian population growth of 1.4% [33].
[ΨΨ]5 years total costs was calculated from year 2021 to 2025.

Discussion

We created a decision tree framework to evaluate the cost-effectiveness of the pneumococcal vaccine, PPV23 for Malaysian Hajj pilgrims compared to the current status of no vaccination. The results presented here demonstrated that the implementation of a PPV23 vaccination for Malaysian hajj pilgrims can effectively reduce health (cases averted) and economic burden (cost savings) associated with pneumococcal diseases. Under the base case scenario, PPV23 could be considered highly cost-effective, with an ICER of MYR -449.3 per case averted. This is in line with other modelling studies conducted in several countries like the Netherlands, Belgium, Turkey, Colombia and Brazil also found PPV23 to be a cost-effective intervention [17,34-37]. An additional 110,966 cases of the pneumococcal disease could be averted with an estimated MYR 59.6 million cost-saving. This is in line with other studies, which reported the cost-saving intervention [17, 37-40]. However, further comparison with the international literature is not possible due to different assumptions on model structures, as well as parameters included such as time horizon, vaccine price, vaccine effectiveness and incidence of related pneumococcal disease.

The hypothetical analysis conducted in this study shows that the implementation of the PPV23 vaccine to a 2017 cohort of Malaysian Hajj pilgrims has the potential to reduce the pneumococcal disease burden by 56.8%. The costs to vaccinate a cohort of Malaysian Hajj pilgrims (MYR8.8 million) are much smaller than the medical costs of pneumococcal disease (MYR18 million). In this base case analysis, the estimated cost associated with pneumococcal disease was reduced by 46.8%. Due to the unavailable cost of PPV23, the best available cost of vaccine in the region was used in the analysis [31]. This may potentially produce variability with the data included in the model. Hence, to ascertain a programme is still a cost-effective option, uncertainty analysis (one-way and probabilistic sensitivity) was conducted. This implying that a relevant variation of the input parameters would not alter the conclusions of this cost-effectiveness analysis study. The important uncertainties are presented through one-way sensitivity analysis, which showed that the probability of getting the pneumococcal disease without vaccination affects ICER the most followed by the probability of septicemia. Most notably, the PPV23 vaccination cost is also a major player driving the ICERs. Certainly, the current cost of vaccine could potentially avoid large numbers of pneumococcal diseases

and regard as a cost-saving measure. Besides, probabilistic sensitivity analyses showed that ICERs were predominantly cost-effective, with a very small percentage of ICERs being dominated.

The introduction of the PPV23 vaccine has increased interest in preventing pneumococcal disease. In most developed countries, the PPV23 is recommended for elderly adults [41-43]. This could be due to its broad serotype coverage, which has been estimated to be 84% among adults [44]. In relation to hajj, recommendation on the uptake of PPV23 vaccine prior to performing Hajj has been evolved in certain countries, including in Malaysia. However, there is a lack of consensus as to whether the recommendation of the PPV23 is for all adults or specifically for the Hajj population. In this analysis, we focused on the PPV23 intervention among Hajj pilgrims, which normally dominate by the elderly population. According to a study conducted by Eilers et al. [45], pneumococcal disease is considered the most severe and most relevant disease to vaccinate among the elderly. Perhaps, assumption of a 100% uptake of PPV23 among the Hajj population in this study could be considered as a potential strategy to vaccinate Malaysians who wish to perform hajj in the future. Although this study did not consider payment options from the Hajj pilgrims or co-payment between the government and Hajj pilgrims, full subsidisation from the Malaysian government could still offer a cost-saving measure and ultimately reduce disease and economic burden from a healthcare system perspective.

To the authors' knowledge, this is the first to look into the cost-effectiveness of implementing pneumococcal vaccination using the PPV23 vaccine, among Malaysian Hajj pilgrims, in terms of both health outcomes and budget impact. Our study has certain strengths. The PPV23 effectiveness was based on the prospective cohort that involved vaccinated and a non-vaccinated group of Malaysian Hajj pilgrims in 2015 [24], which clearly showed that PPV23 is efficacious in the prevention of pneumococcal pneumonia. Besides, findings from the budget impact analysis enable the policymakers to know the total budget, incremental costs, and costs saving by implementing the PPV23 vaccination. This will allow improving the allocation of scarce healthcare resources and would facilitate the practice of evidence-based reimbursement decision making. Our findings also add to the limited evidence available regarding the cost-effectiveness of pneumococcal vaccination among Hajj pilgrims. This could be of help to contribute to the knowledge generation of future pneumococcal vaccination

programmes among the hajj population. We conducted this study from the provider perspective, which the medical costs incurred by the Malaysian Government. This study perhaps could provide useful information to policymakers for the planning and development of a PPV23 vaccination programme for Malaysian Hajj pilgrims. It could also be used to allocate scarce resources for the health of Hajj pilgrims, which includes qualified personnel and operational costs of hospitals and clinics in Saudi.

Notwithstanding a thorough analysis, this study still experienced some limitations. First, data used in this study were subjected to availability and the analyses were restricted based on the best available data shared. The data inputs into the model were obtained from the literature (non-Malaysian population), which is confined to Hajj pilgrims or the elderly. This is due to the absence of published local data and potentially makes estimates in the model uncertain. However, the one-way and probabilistic sensitivity analyses were conducted to overcome this constraint using a plausible range of uncertain parameters. Second, the time frame or duration of this study was set at one year, within the year 2017 with no further follow up of the Hajj pilgrims, as the pilgrimage typically occurs only once a year. Third, only the operational costs at Saudi Arabia health facilities were considered and included in this cost-effectiveness analysis, thus, post-hajj operational costs are excluded, given that Hajj pilgrims may develop symptoms and seek treatment after they returned to Malaysia. Besides, the cost-effectiveness results were estimated using case averted, which further limit the comparison to the WTP threshold and with other studies incorporated an estimation using DALYs or QALYs. Lastly, the context of this study was confined to a one-off exposure to streptococcal infection within the specified time frame. For this, herd immunity was not included at the data analysis stage because the evidence on this topic was not available, particularly in a study in which vaccination was only given to a relatively small population such as the Hajj pilgrims. Despite these shortcomings, our analysis provides enlightenment into the potential of PPV23 to reduce the burden of pneumococcal disease.

Conclusions

This study assesses the cost-effectiveness and budget impact of a PPV23 vaccination programme for Malaysian Hajj pilgrims, compared with no vaccination. The results presented

here demonstrated that PPV23 would be a cost-effective measure to reduce the economic and health burden of pneumococcal disease. Besides, it is suggested that this vaccination programme would avoid a significant number of pneumococcal cases over 5 years and would be a cost-saving measure from a health system perspective. At the current rate, it is evident that the health and economic needs of the Hajj population pool will be enormous. In particular, costs related to pneumococcal disease treatment for this specific group will be more demanding in the future. Certainly, this necessitates a cost-effective and efficient approach. Thus, on the basis of the study findings, it could be suggested that PPV23 should be implemented as the vaccination programme for Malaysian performing the hajj, especially those at risk of pneumococcal disease.

Acknowledgements

The authors wish to thank the Director-General of Health, Malaysia that made this publication possible. We also acknowledge Lembaga Tabung Haji Malaysia and the Institute for Medical Research, Ministry of Health Malaysia for providing data for this study. We would like to thank the reviewers for their thoughtful comments and suggestions.

References

1. Memish ZA, Zumla A, Alhakeem RF, Assiri A, Turkestani A, Al Harby KD, et al. Hajj: infectious disease surveillance and control. Lancet. 2014;383(9934):2073–82.

2. AlBarrak A, Alotaibi B, Yassin Y, Mushi A, Maashi F, Seedahmed Y, et al. Proportion of adult community-acquired pneumonia cases attributable to Streptococcus pneumoniae among Hajj pilgrims in 2016. Int J Infect Dis. 2018;69:68–74.

3. Shirah BH, Zafar SH, Alferaidi OA, Sabir AMM. Mass gathering medicine (Hajj Pilgrimage in Saudi Arabia): The clinical pattern of pneumonia among pilgrims during Hajj. J Infect Public Health. 2017;10(3):277–86.

4. Al-Tawfiq JA, Memish ZA. Prevention of pneumococcal infections during mass gathering. Hum Vaccin Immunother. 2016;12(2):326–30.

5. Mandourah Y, Al-Radi A, Ocheltree AH, Ocheltree SR, Fowler RA. Clinical and temporal patterns of severe pneumonia causing critical illness during Hajj. BMC Infect Dis. 2012;12(1):117.

6. Lynch JP 3rd, Zhanel GG. Streptococcus pneumoniae: epidemiology, risk factors, and strategies for prevention. Semin Respir Crit Care Med. 2009;30(2):189–209.

7. Parsons HK, Dockrell DH. The burden of invasive pneumococcal disease and the potential for reduction by immunisation. Int J Antimicrob Agents. 2002;19(2):85–93.

8. Ebrahim SH, Memish ZA, Uyeki TM, Khoja TAM, Marano N, McNabb SJN. Public health. Pandemic H1N1 and the 2009 hajj. Science. 2009;326(5955):938–40.

9. Benkouiten S, Gautret P, Belhouchat K, Drali T, Salez N, Memish ZA, et al. Acquisition of Streptococcus pneumoniae carriage in pilgrims during the 2012 Hajj. Clin Infect Dis. 2014;58(4):e106-9.

10. Alzahrani AS, Alhumaidi F, Altowairqi A, Al-Malki W, AlFadhli I. Screening for cognitive impairment in Arabic-speaking Hajj pilgrims. Egypt J Neurol Psychiatr Neurosurg [Internet]. 2019;55(1). Available from: http://dx.doi.org/10.1186/s41983-019-0111-x

11. Alharbi NS, Al-Barrak AM, Al-Moamary MS, Zeitouni MO, Idrees MM, Al-Ghobain MO, et al. The Saudi Thoracic Society pneumococcal vaccination guidelines-2016. Ann Thorac Med. 2016;11(2):93–102.

12. Leventer-Roberts M, Feldman BS, Brufman I, Cohen-Stavi CJ, Hoshen M, Balicer RD. Effectiveness of 23-valent pneumococcal polysaccharide vaccine against invasive disease and hospital-treated pneumonia among people aged ≥65 years: a retrospective case-control study. Clin Infect Dis. 2015;60(10):1472–80.

13. Rafferty ERS, Gagnon HL, Farag M, Waldner CL. Economic evaluations of vaccines in Canada: a scoping review. Cost Eff Resour Alloc. 2017;15(1):7.

14. van de Vooren K, Duranti S, Curto A, Garattini L. Cost effectiveness of the new pneumococcal vaccines: a systematic review of European studies. Pharmacoeconomics. 2014;32(1):29–45.

15. Wolff E, Storsaeter J, Örtqvist Å, Naucler P, Larsson S, Lepp T, et al. Cost-effectiveness of pneumococcal vaccination for elderly in Sweden. Vaccine. 2020;38(32):4988–95.

16. Thorrington D, van Rossum L, Knol M, de Melker H, Rümke H, Hak E, et al. Impact and cost-effectiveness of different vaccination strategies to reduce the burden of pneumococcal disease among elderly in the Netherlands. PLoS One. 2018;13(2):e0192640.

17. De Graeve D, Lombaert G, Goossens H. Cost-effectiveness analysis of pneumococcal vaccination of adults and elderly persons in Belgium. Pharmacoeconomics. 2000;17(6):591–601.

18. Ahmed QA, Arabi YM, Memish ZA. Health risks at the Hajj. Lancet. 2006;367(9515):1008–15.

19. Al-Ghamdi SM, Akbar HO, Qari YA, Fathaldin OA, Al-Rashed RS. Pattern of admission to hospitals during muslim pilgrimage (Hajj). Saudi Med J. 2003;24(10):1073–6.

20. Deris ZZ, Hasan H, Sulaiman SA, Wahab MSA, Naing NN, Othman NH. The prevalence of acute respiratory symptoms and role of protective measures among Malaysian hajj pilgrims. J Travel Med. 2010;17(2):82–8.

21. Balkhy HH, Memish ZA, Bafaqeer S, Almuneef MA. Influenza a common viral infection among Hajj pilgrims: time for routine surveillance and vaccination. J Travel Med. 2004;11(2):82–6.

22. Feldman C, Abdulkarim E, Alattar F, Al Lawati F, Al Khatib H, Al Maslamani M, et al. Pneumococcal disease in the Arabian Gulf: recognizing the challenge and moving toward a solution. J Infect Public Health. 2013;6(6):401–9.

23. Health conditions for travellers to Saudi Arabia for the pilgrimage to Mecca (Hajj), 2016. (2016). *Releve epidemiologique hebdomadaire*, *91*(26-27), 331–335.

24. Aljunid S, Maimaiti N, Ahmed Z, Muhammad Nur A, Md Isa Z, Azmi S, et al. Economic impact of pneumococcal protein-D conjugate vaccine (PHiD-CV) on the Malaysian National Immunization Programme. Value Health Reg Issues. 2014;3:146–55.

25. Yusoff AF. Risk of acquiring pneumonia among the Malaysian hajj pilgrims vaccinated with 23 valent pneumococcal vaccine. Medical Journal Malaysia. 2017;72(1):75.

26. Domingo P, Pomar V, de Benito N, Coll P. The spectrum of acute bacterial meningitis in elderly patients. BMC Infect Dis. 2013;13(1):108.

27. Alzahrani AG, Choudhry AJ, Al Mazroa MA, Turkistani AHM, Nouman GS, Memish ZA. Pattern of diseases among visitors to Mina health centers during the Hajj season, 1429 H (2008 G). J Infect Public Health. 2012;5(1):22–34.

28. Baharoon S, Al-Jahdali H, Al Hashmi J, Memish ZA, Ahmed QA. Severe sepsis and septic shock at the Hajj: etiologies and outcomes. Travel Med Infect Dis. 2009;7(4):247–52.

29. Razavi SM, Mardani M, Salamati P. Infectious diseases and preventive measures during hajj mass gatherings: A review of the literature. Arch Clin Infect Dis [Internet]. 2018;13(3). Available from: http://dx.doi.org/10.5812/archcid.62526

30. GDP per capita, PPP (current international $) [Internet]. Worldbank.org. [cited 2021 Aug 24]. Available from: https://data.worldbank.org/indicator/NY.GDP.PCAP.PP.CD

31. Mo X, Gai Tobe R, Liu X, Mori R. Cost-effectiveness and health benefits of pediatric 23-valent pneumococcal polysaccharide vaccine, 7-valent pneumococcal conjugate vaccine and forecasting 13-valent pneumococcal conjugate vaccine in China. Pediatr Infect Dis J. 2016;35(11):e353–61.

32. Lim YW, Shafie AA, Chua GN, Ahmad Hassali MA. Determination of cost-effectiveness threshold for health care interventions in Malaysia. Value Health. 2017;20(8):1131–8.

33. Worldbank.org. [cited 2021 Aug 24]. Available from: https://data.worldbank.org/indicators/SP.POP.GROW

34. Baltussen R. Cost-effectiveness of vaccination against pneumococcal pneumonia in The Netherlands. Eur J Public Health. 1997;7(2):153–61.

35. Akin L, Kaya M, Altinel S, Durand L. Cost of pneumococcal infections and cost-effectiveness analysis of pneumococcal vaccination at risk adults and elderly in Turkey. Hum Vaccin. 2011;7(4):441–50.

36. Castañeda-Orjuela C, Alvis-Guzmán N, Paternina AJ, De la Hoz-Restrepo F. Cost-effectiveness of the introduction of the pneumococcal polysaccharide vaccine in elderly Colombian population. Vaccine. 2011;29(44):7644–50.

37. Neto JT, de Araujo GTB, Gagliardi A, Pinho A, Durand L, Fonseca M. Cost-effectiveness analysis of pneumococcal polysaccharide vaccination from age 60 in São Paulo State, Brazil. Hum Vaccin. 2011;7(10):1037–47.

38. Sisk JE, Moskowitz AJ, Whang W, Lin JD, Fedson DS, McBean AM, et al. Cost-effectiveness of vaccination against pneumococcal bacteremia among elderly people. JAMA. 1997;278(16):1333–9.

39. Sisk JE, Riegelman RK. Cost effectiveness of vaccination against pneumococcal pneumonia: an update. Ann Intern Med. 1986;104(1):79–86.

40. Plans P. Coste-efectividad de la vacunación antineumocócica 23-valente en Cataluña. Gac Sanit. 2002;16(5):392–400.

41. Pebody RG, Hippisley-Cox J, Harcourt S, Pringle M, Painter M, Smith G. Uptake of pneumococcal polysaccharide vaccine in at-risk populations in England and Wales 1999-2005. Epidemiol Infect. 2008;136(3):360–9.

42. Hull B, Dey A, Campbell-Lloyd S, Menzies RI, McIntyre PB. NSW annual immunisation coverage report, 2010. N S W Public Health Bull. 2011;22(9–10):179–95.

43. Hull B, Dey A, Campbell-Lloyd S, Menzies RI, McIntyre PB. NSW annual immunisation coverage report, 2011. N S W Public Health Bull. 2012;23(9–10):179–86.

44. Al-Mazrou A, Twum-Danso K, Al Zamil F, Kambal A. Streptococcus pneumoniae serotypes/serogroups causing invasive disease in Riyadh, Saudi Arabia: extent of coverage by pneumococcal vaccines. Ann Saudi Med. 2005;25(2):94–9.

45. Eilers R, Krabbe PFM, de Melker HE. Attitudes of Dutch general practitioners towards vaccinating the elderly: less is more? BMC Fam Pract. 2015;16(1):158.

Figure 1

Click here to access/download;Figure;Fig1.tiff

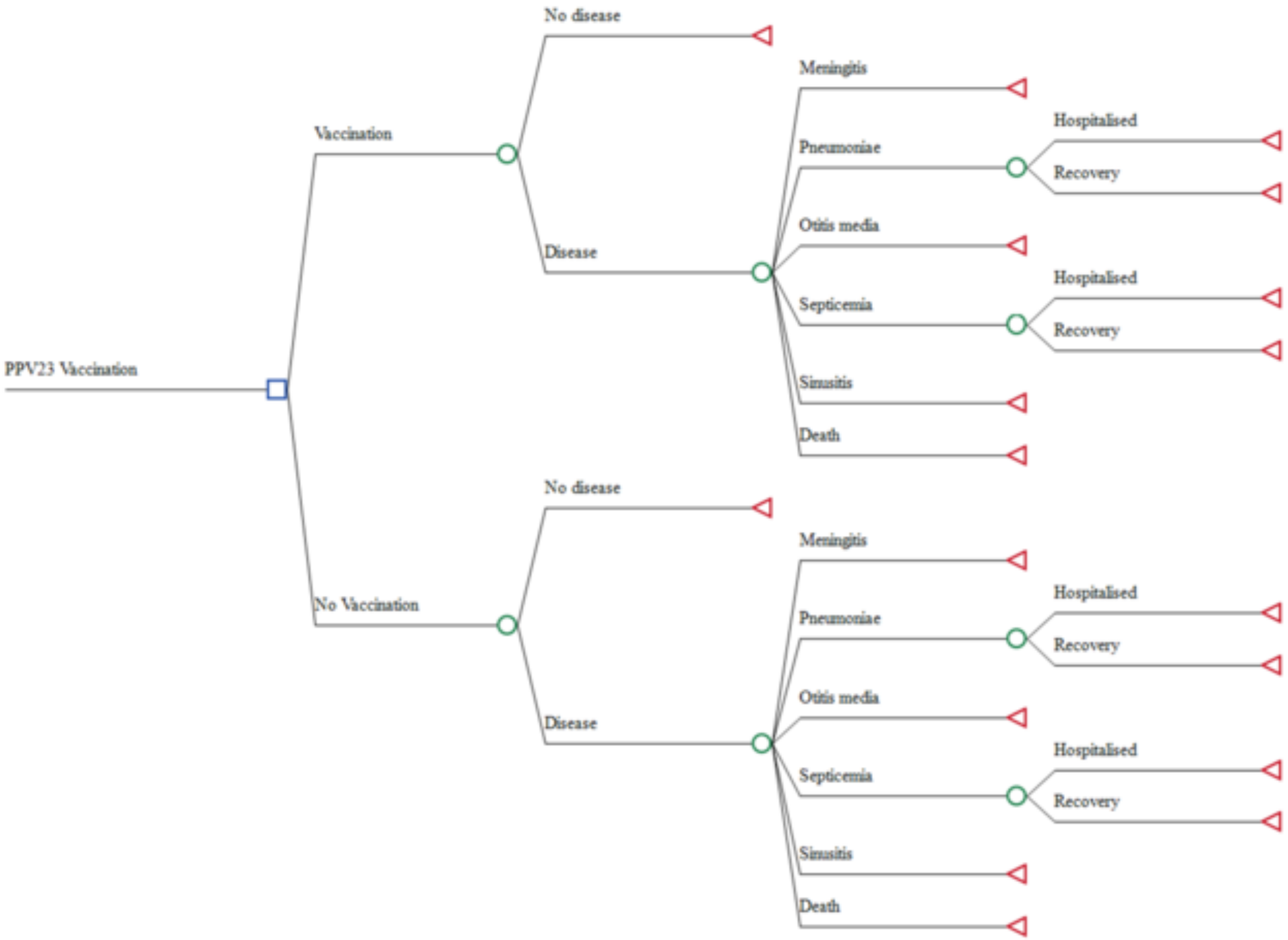

Figure 2

Click here to access/download;Figure;Fig2.tiff

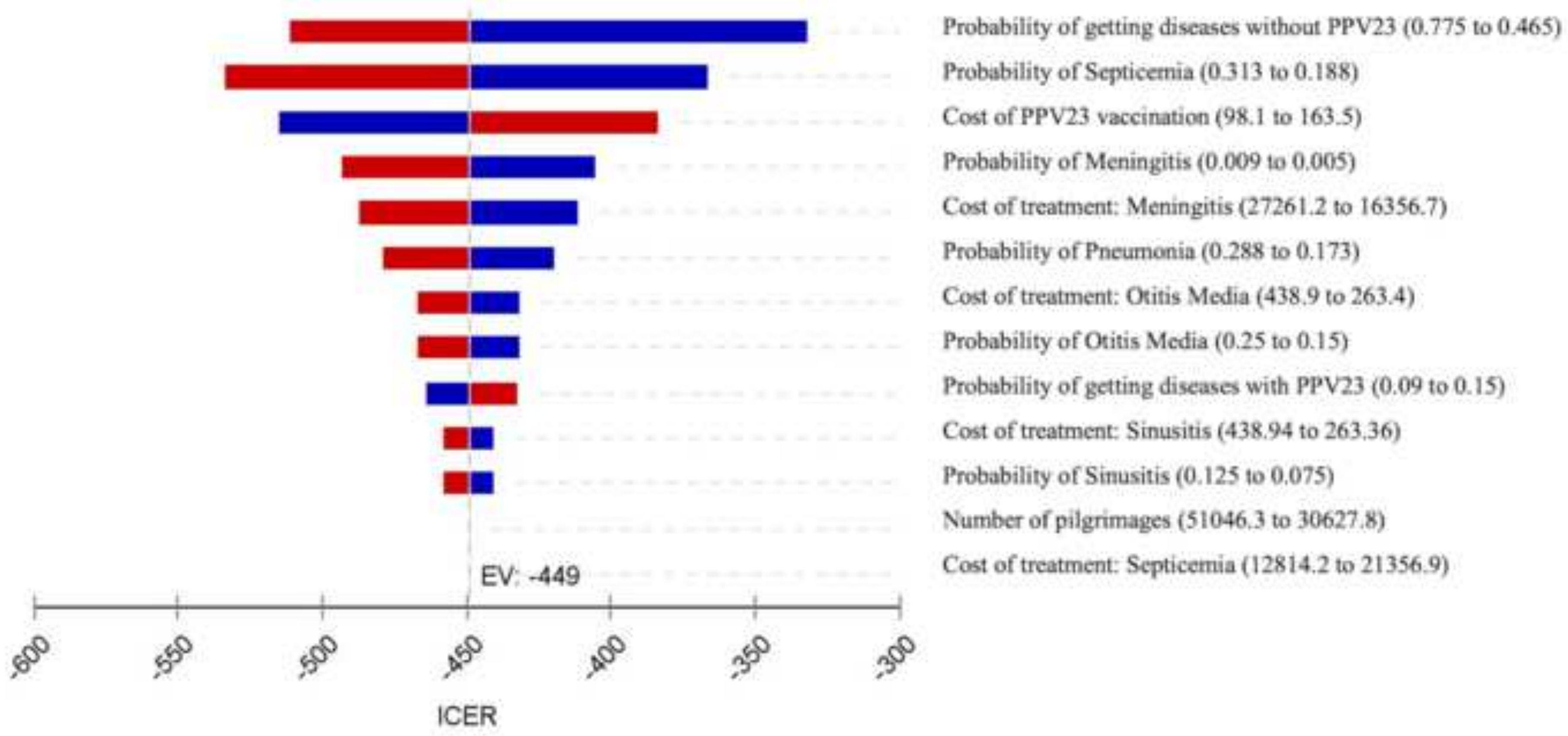

Figure 3

Click here to access/download;Figure;Fig3.tiff

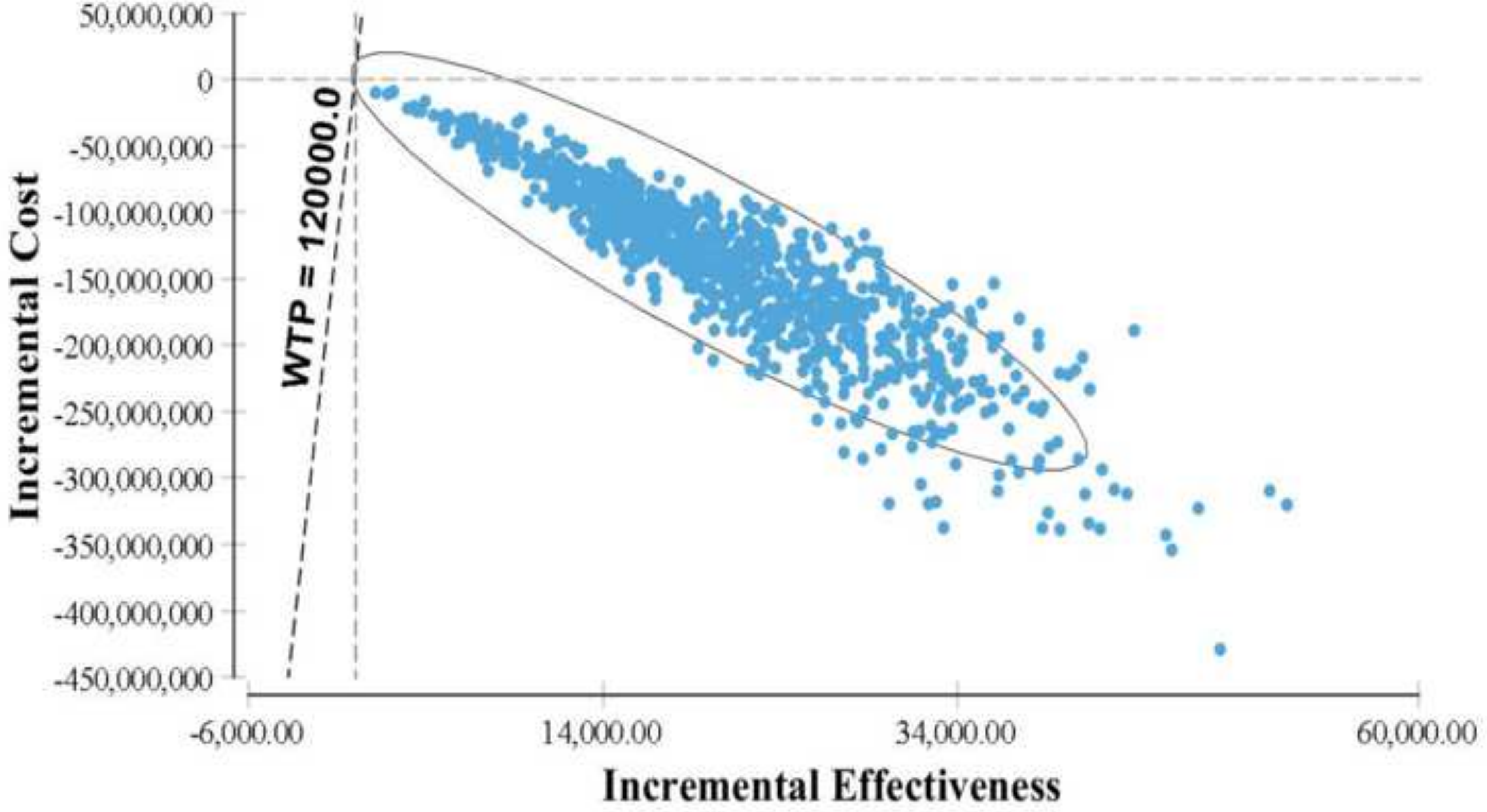

Figure 4

Click here to access/download;Figure;Fig4.tiff

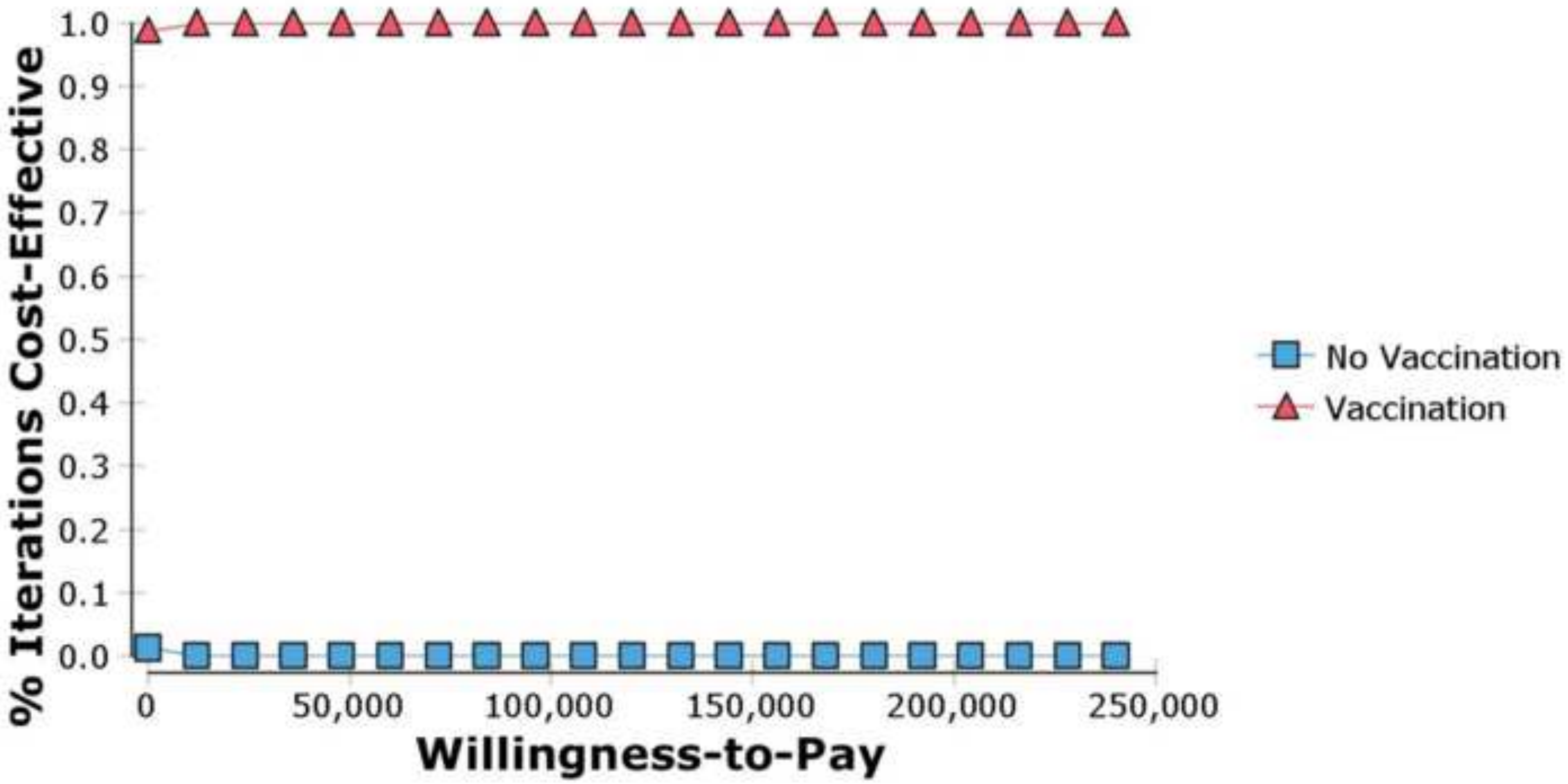

Supplement: Attachment — Submitted filename: PONE-D-21-27712_reviewer.pdf [file pone.0262949.s001.pdf]
