# Supplementary material for: Cost-effectiveness and budget impact analysis of PPV23 vaccination for the Malaysian Hajj pilgrims
Source: PLoS One. 2022 Jan 24;17(1):e0262949. doi: 10.1371/journal.pone.0262949 (PMC8786116; doi:10.1371/journal.pone.0262949)
Supplement: Attachment — Submitted filename: Response to Reviewers.docx [file pone.0262949.s002.docx]

**Response to Reviewers**

Dear Professor Mahmud Khan,

Thank you for giving us the opportunity to submit a revised draft of the manuscript “Cost-effectiveness and budget impact analysis of PPV23 vaccination for the Malaysian Hajj pilgrims”. We appreciate the time and effort that you and the reviewers dedicated to providing feedback on our manuscript and are grateful for the insightful comments. We have revised the manuscript accordingly (incorporate all suggestions) and provided specific answers below, written in red font. All changes made in the main document are marked using track changes.

Academic editor:

1. Please ensure that your manuscript meets PLOS ONE’s style requirements, including those for file naming.

*We have thoroughly checked the manuscript and prepared it following PLOS ONE’s style requirements.*

2. Please include your full ethics statement in the ‘Methods’ section of your manuscript file. In your statement, please include the full name of the IRB or ethics committee who approved or waived your study, as well as whether or not you obtained informed written or verbal consent. If consent was waived for your study, please include this information in your statement as well.

*We have added a full ethics statement in the Methods section line 251. In this statement, the ethics committee is mentioned together with informed consent which is not necessary for this study.*

3. Please review your reference list to ensure that it is complete and correct. If you have cited papers that have been retracted, please include the rationale for doing so in the manuscript text, or remove these references and replace them with relevant current references.

*All included references were checked for completeness and correctness. There are no retracted papers cited in this study.*

Reviewers:

Reviewer#1

This work results will be helpful not just to the Malaysian Hajj Visitors, but to all Hajj visitors from other countries. The only thing that I need you to review please is the vaccination required by the Saudi government which change every year, and different from country to a country based on the public health status of the visitor country.

*We have reviewed and added several health requirements that must be met by Hajj pilgrims from different countries, where the health regulations are set by the Saudi Ministry of Health (Refer to introduction section, lines 108-117). This information can be obtained from the Embassy of the Kingdom of Saudi Arabia.*

Reviewer#2

The manuscript has been prepared in accordance with the results of the study and can be concluded well. For the writing of the words, Hajj and Pilgrims should be consistent, some are written Hajj Pilgrims, some are written Hajj only or Pilgrims only.

*We go through the manuscript and the word ‘pilgrims’ won’t appear alone instead, written as Hajj pilgrims. And wherever relevant, the word hajj could be appeared alone according to the context of the sentences. (Hajj pilgrims are referred to as the Muslim population while hajj is the rituals performed by these groups).*

For the figure, the decision tree model doesn’t appear, I don’t know if there’s a difference between the script I received and the original.

*We apologise for the inconvenience caused. However, I guarantee the decision tree figure that you received is similar to the original one. The tree model was created based on a prior study conducted by Aljunid et al., 2014. A slight modification of the model was made based on data availability which was confined to the Hajj pilgrims population.*

In my opinion, the description on probabilistic sensitivity analysis is incomplete and unclear, so it needs to be described properly and can be added with reference materials related to this topic.

*Changes were made to the description of probabilistic sensitivity analysis (refer lines 234-241) to make it clear and understandable.*
